# Anaplastic Medullary Ependymoma Presenting as Subarachnoid Hemorrhage

**DOI:** 10.1155/2013/701820

**Published:** 2013-03-06

**Authors:** Nicolas Nicastro, Armin Schnider, Béatrice Leemann

**Affiliations:** Neurorehabilitation Service, Department of Clinical Neurosciences, Geneva University Hospital, Avenue de Beau-Séjour 26, 1206 Geneva, Switzerland

## Abstract

A-41-year old man presented with violent thunderclap headache and a bilateral proprioceptive sensibility deficit of the upper limbs. Cerebral CT scan and MRI were negative. Lumbar puncture confirmed subarachnoid hemorrhage (SAH), but cerebral angiography was negative. Three months later, the patient presented with paraparesis, and a thorough work-up revealed a diffuse, anaplastic extramedullary C7-D10 ependymoma with meningeal carcinomatosis considered the source of hemorrhage. The patient went through a D5-D8 laminectomy, temozolomide chemotherapy, and radiotherapy. The situation remained stable for a few months. In this paper, we would like to emphasize that spinal masses should be considered in cases of SAH with negative diagnostic findings for aneurysms or arteriovenous malformation.

## 1. Introduction

Subarachnoid hemorrhage (SAH) is normally due to intracranial lesions (mostly aneurysms of vascular malformation). SAH due to spinal lesions is less common and mostly a consequence of a spinal trauma or arteriovenous malformation [1, 2]. Spinal masses account for a very small minority of SAH and include ependymoma, meningioma, metastasis, or hemangioblastoma [3]. We consider this case important, as it may propose a clearer strategy concerning the work-up for subarachnoid hemorrhage. Lumbar puncture remains a useful diagnostic tool when initial CT scan does not show any SAH. In addition, investigations for spinal masses must be performed with negative cerebral angiography.

## 2. Case Presentation

A-41-year old man with no particular medical history presented with severe thunderclap headache. He had no photo-phonophobia and no vomiting. He did not notice any visual phenomenon or motor or coordination impairment.

Cognitive assessment was unremarkable; cranial nerves and motor and sensory examination was normal, except for a bilateral proprioceptive deficit of the upper limbs. The patient experienced mild imbalance on the Romberg maneuver.

SAH was suspected and a cranial CT Scan was performed, which was negative, as well as a brain MRI. However, a lumbar puncture with a three-tube test revealed xanthochromic cerebrospinal fluid, 260 red blood cells, and 6 leukocytes.

**Figure 1:**
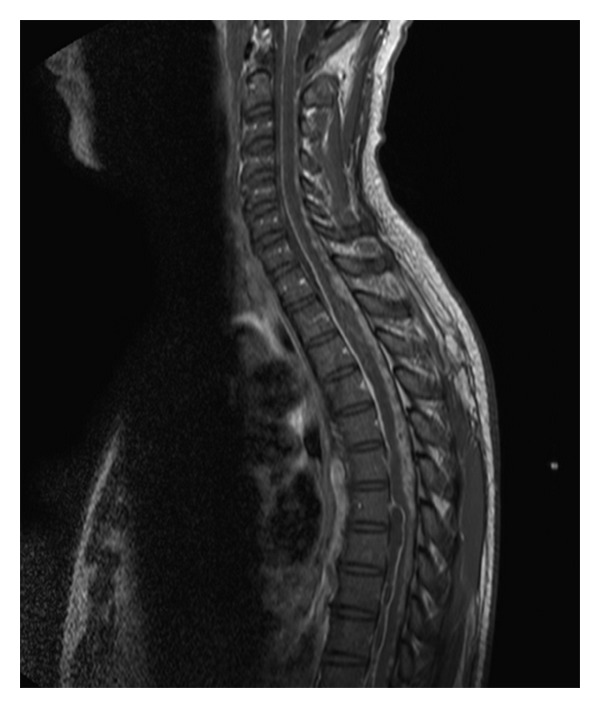
Whole spine T1-weighted MRI with diffuse intradural infiltration of the ependymoma.

**Figure 2:**
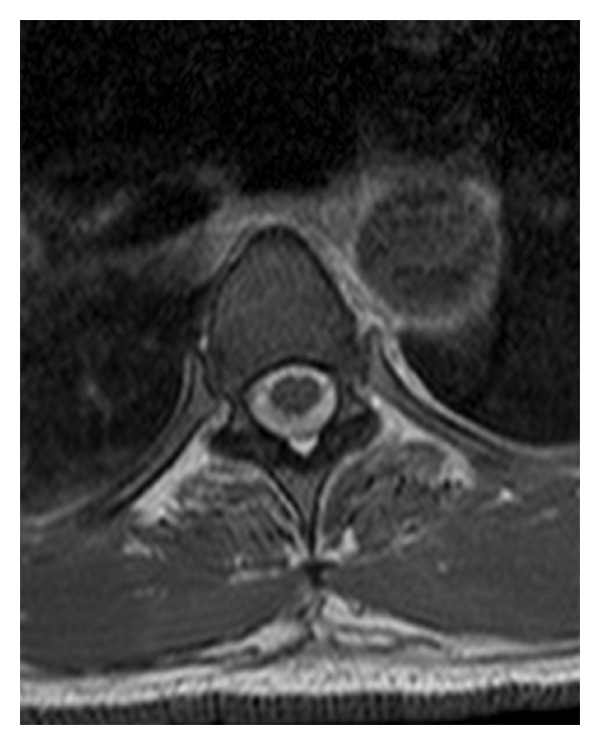
Axial T1-weighted dorsal MRI showing diffuse infiltration of the tumor.

**Figure 3:**
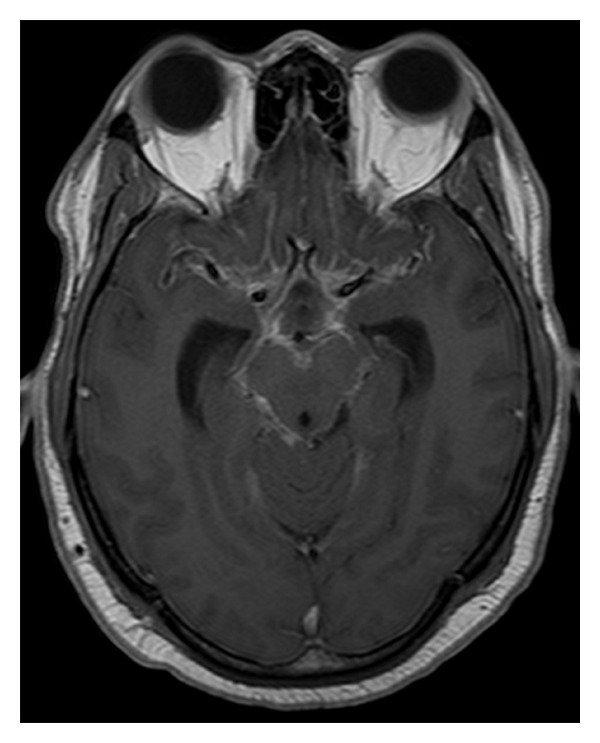
Cerebral T1-weighted MRI showing diffuse leptomeningeal carcinomatosis.

Cerebral angiography was later performed and revealed no vascular malformation or cerebral aneurysm. At this point, the patient went asymptomatic and was discharged with a diagnosis of undetermined regressive headache. Two months later, the patient presented again to the emergency room with severe headache. Brain CT scan showed ventricular dilation; a ventriculoperitoneal shunt was inserted. The patient progressively resented low back pain and lower limb weakness, as well as fecal and urinary incontinence. MRI of the spinal column showed a diffuse heterogenous intradural perimedullary infiltration from the bulb to the lumbar spine, predominantly on the C7-D10 levels, associated with an intramedullary D1-D3 extension (Figures 1 and 2) and a leptomeningeal carcinomatosis (Figure 3). On the D6-D10 levels, the spinal cord was pushed back by a liquid premedullary collection. 

A D5-D8 laminectomy with partial decompressive resection was performed and the histopathological examination of the specimen showed an anaplastic (grade III) ependymoma with a 10% Mib1. A rescue chemotherapy (temozolomide) was begun as well as craniospinal radiotherapy and steroids.

**Figure 4:**
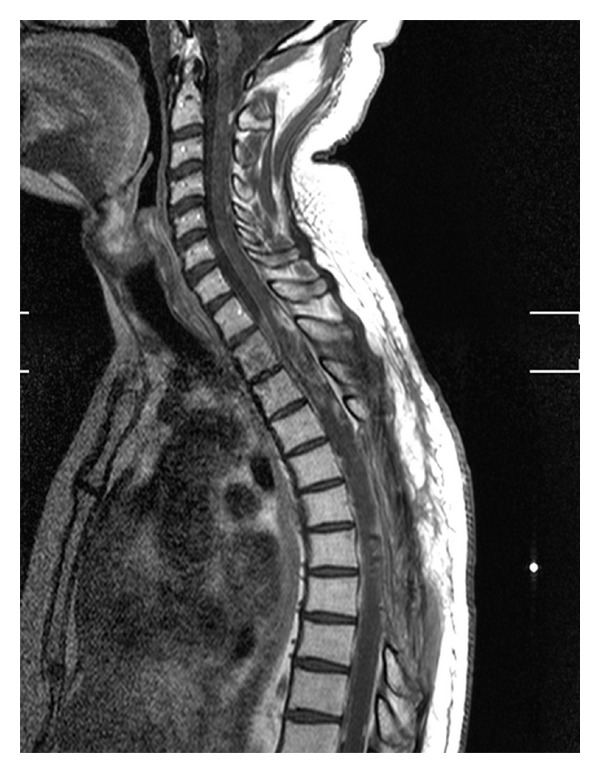
Posttreatment cervicodorsal MRI: we can notice a remarkable improvement of the spinal infiltration.

**Figure 5:**
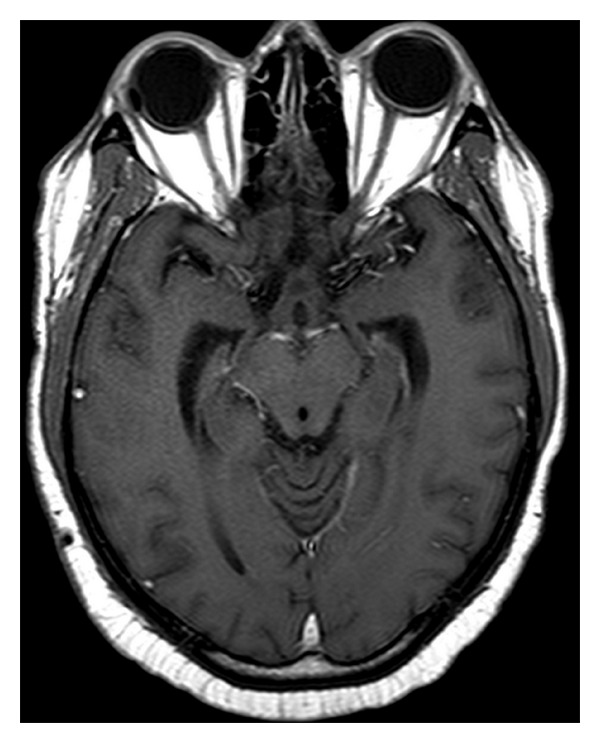
Cerebral T1-weighted MRI posttreatment: the carcinomatosis disappeared.

Control MRI shows a remarkable improvement of the spinal lesions and the disappearance of the meningeal carcinomatosis (Figures 4 and 5). 

The patient remained stable for 8 months and then returned to Nigeria. We do not have any news concerning his current state.

## 3. Discussion 

In this paper, we would like to emphasize the importance of investigating the spinal column in the context of a subarachnoid hemorrhage with negative cerebral angiography, especially when it presents with an insidious, prolonged history. As stated by Little et al. [4], repeat angiography is a useful diagnostic modality, as it can reveal a thrombosed or a less than 5 mm aneurysm. MRI, especially FLAIR sequences, can also be useful to detect convexity hemorrhage in patients with negative CT scan and positive lumbar puncture. Finally, cervico-dorso-lumbar MRI is a rewarding approach to detect spinal vascular or neoplastic lesions. As Ulrich et al. [5] demonstrated in their study of 18 ependymoma presenting as SAH, most of them are myxopapillary ependymoma of the lumbar spine or the conus medullaris [6]. As a matter of fact, a work-up for spinal axis should always be considered as a further investigation for SAH with negative cerebral angiography.
